# Sex Differences in the Clinical Presentation and Natural History of Dilated Cardiomyopathy

**DOI:** 10.1016/j.jchf.2023.10.009

**Published:** 2024-02

**Authors:** Ruth Owen, Rachel Buchan, Michael Frenneaux, Julian W.E. Jarman, Resham Baruah, Amrit S. Lota, Brian P. Halliday, Angharad M. Roberts, Cemil Izgi, Harriette G.C. Van Spall, Erin D. Michos, John J.V. McMurray, James L. Januzzi, Dudley J. Pennell, Stuart A. Cook, James S. Ware, Paul J. Barton, John Gregson, Sanjay K. Prasad, Upasana Tayal

**Affiliations:** a Department of Medical Statistics, London School of Hygiene and Tropical Medicine, London, United Kingdom; b National Heart Lung Institute, Imperial College London, United Kingdom; c Royal Brompton & Harefield Hospitals, Guy’s and St. Thomas’ NHS Foundation Trust, London, United Kingdom; d Department of Medicine, Department of Health Research Methods, Evidence, and Impact, McMaster University, Hamilton, Canada; e Cardiology Division, Massachusetts General Hospital, Baim Institute for Clinical Research, Boston, Massachusetts, USA; f Division of Cardiology, Johns Hopkins University School of Medicine, Baltimore, Maryland, USA; g British Heart Foundation Cardiovascular Research Centre, University of Glasgow, Glasgow, United Kingdom; h MRC London Institute of Medical Sciences, London, United Kingdom

**Keywords:** females, heart, males, sex

## Abstract

**Background:**

Biological sex has a diverse impact on the cardiovascular system. Its influence on dilated cardiomyopathy (DCM) remains unresolved.

**Objectives:**

This study aims to investigate sex-specific differences in DCM presentation, natural history, and prognostic factors.

**Methods:**

The authors conducted a prospective observational cohort study of DCM patients assessing baseline characteristics, cardiac magnetic resonance imaging, biomarkers, and genotype. The composite outcome was cardiovascular mortality or major heart failure (HF) events.

**Results:**

Overall, 206 females and 398 males with DCM were followed for a median of 3.9 years. At baseline, female patients had higher left ventricular ejection fraction, smaller left ventricular volumes, less prevalent mid-wall myocardial fibrosis (23% vs 42%), and lower high-sensitivity cardiac troponin I than males (all *P* < 0.05) with no difference in time from diagnosis, age at enrollment, N-terminal pro–B-type natriuretic peptide levels, pathogenic DCM genetic variants, myocardial fibrosis extent, or medications used for HF. Despite a more favorable profile, the risk of the primary outcome at 2 years was higher in females than males (8.6% vs 4.4%, adjusted HR: 3.14; 95% CI: 1.55-6.35; *P* = 0.001). Between 2 and 5 years, the effect of sex as a prognostic modifier attenuated. Age, mid-wall myocardial fibrosis, left ventricular ejection fraction, left atrial volume, N-terminal pro–B-type natriuretic peptide, high-sensitivity cardiac troponin I, left bundle branch block, and NYHA functional class were not sex-specific prognostic factors.

**Conclusions:**

The authors identified a novel paradox in prognosis for females with DCM. Female DCM patients have a paradoxical early increase in major HF events despite less prevalent myocardial fibrosis and a milder phenotype at presentation. Future studies should interrogate the mechanistic basis for these sex differences.

Sex is understood to have a diverse impact on the cardiovascular system in both health and disease. Despite an evolving understanding of how sex may affect manifestation and progression of cardiovascular disease, its influence on the morpho-functional and clinical manifestations of dilated cardiomyopathy (DCM), one of the commonest causes of heart failure (HF), remains unresolved.

DCM affects up to 1 in 250 people. The etiology can be genetic, environmental, immune, or idiopathic.^1^^,^^2^ It is a growing medical and economic burden on health care systems. Despite therapeutic advances for DCM, outcomes are poor.^3^^,^^4^ There is a pressing need to understand the contributors to disease to improve prognosis. Understanding biological sex as a modifier of cardiomyopathy is a major unmet need in the field as identified by key guidelines and commissions.^5^, ^6^, ^7^

In this prospective observational study of well-characterized individuals with DCM, we sought to characterize morpho-functional differences and contemporary outcomes in DCM by sex and evaluate major DCM prognostic factors of left ventricular ejection fraction (LVEF) and mid-wall myocardial fibrosis in the context of sex. Understanding sex differences in phenotype and prognosis offers potential to improve outcomes through tailored diagnostic and therapeutic strategies.

## Methods

### Study cohort

Participants comprised 604 patients with a clinical diagnosis of DCM confirmed by late gadolinium–enhanced (LGE) cardiac magnetic resonance (CMR) who were prospectively enrolled in the Royal Brompton Hospital Cardiovascular Research Centre Biobank project between 2009 and 2015. This is one of the largest and most comprehensively phenotyped cohorts of patients with DCM. Study participants were recruited via a broad network of >30 referring hospitals across London and the south of England. Participants were enrolled at the time of their first diagnostic CMR from consecutive referrals to the CMR unit and from cardiology clinic. All patients provided written informed consent. The study was approved by the regional ethics committee (South Central Research Ethics Committee 19/SC/0257).

### DCM diagnosis

The diagnosis of DCM was made based on CMR findings of left ventricular dilation and systolic impairment assessed by LVEF using age, sex, and body surface area adjusted nomograms in line with European Society of Cardiology guidelines.^8^^,^^9^ Individuals with significant coronary artery disease were excluded (>50% stenosis in one or more major epicardial arteries identified on computed tomography or invasive coronary angiography, previous percutaneous coronary intervention, previous coronary artery bypass grafting, or evidence of myocardial infarction on CMR). Other exclusion criteria for DCM included a history of uncontrolled systemic hypertension, chronic excess alcohol consumption meeting criteria for alcoholic cardiomyopathy (>80 g/d for more than 5 years), pericardial disease, congenital heart disease, infiltrative disorders (eg, sarcoidosis), recent acute presentation of myocarditis, or significant primary valvular disease.^10^, ^11^, ^12^ Diabetes or a history of well-controlled hypertension were documented as comorbidities. A contraindication to CMR included the presence of a non-CMR conditional pacemaker, defibrillator, or pacing wires; metal implants (including cochlear or spinal implants, hydrocephalus shunts); vascular clips; or foreign bodies or metal in the eye.

### Cohort characterization

Baseline demographic and clinical information was collected on all patients using patient interview, clinical records, electrocardiograms, and family pedigree data as previously described.^4^^,^^13^ Circulating biomarkers of high-sensitivity cardiac troponin I (hs-cTnI), N-terminal pro–B-type natriuretic peptide (NT-proBNP), high-sensitivity C-reactive protein, and urea and electrolytes were quantified at baseline. The hs-cTnI was measured using chemiluminescent microparticle immunoassay on ARCHITECT i2000SR (Abbott Diagnostics), creatinine was measured using electrochemiluminescence immunoassay on Cobas 8000 c702 modular analyzers (Roche Diagnostics), high-sensitivity C-reactive protein using the Beckman Coulter assay on the AU680 (Beckman Coulter) and NT-proBNP using the Elecsys proBNP II CalSet assay (Roche Diagnostics), all per the manufacturers’ protocols.

All study participants had targeted next-generation genetic sequencing on Illumina or Life Technologies 5500XL platforms using the TruSight Cardio Sequencing kit (Illumina) or a custom SureSelectXT (Agilent) target capture with equivalent content, as previously described.^4^ Variants in the DCM genes titin, *LMNA*, *MYH7*, *TNNT2*, *VCL*, *TPM1*, *TNNC1*, *RBM20*, *DSP*, *BAG3*, *SCN5A,* and *TCAP* were prioritized for analysis. *FLNC* was not available at the time of sequencing. Pathogenic or likely pathogenic genetic variants were grouped into 4 classes: truncating variants in the titin gene (frameshift, nonsense, and essential splice site variants), *LMNA*, other sarcomeric variants, or other DCM variants. All study participants underwent CMR for assessment of cardiac chamber volumes and function and assessment of fibrosis (1.5-T, Siemens Sonata or Avanto scanners, Siemens Medical Systems). Breath-hold steady-state free precession cine images were acquired in 3 long-axis planes and short-axis slices. LGE images were acquired using a breath-hold inversion recovery sequence following administration of 0.1 mmol/kg of gadolinium contrast agent (Magnevist or Gadovist, Bayer). LGE quantification was undertaken on CVI42 (Circle Cardiovascular Imaging Inc) using the full width at half maximum method. Left ventricular (LV) volumes, function, and mass were measured using a semiautomated threshold-based technique (CMRtools, Cardiovascular Imaging Solutions). Maximum left atrial (LA) volumes were assessed from the 2- and 4-chamber views at ventricular end-systole. All volume and mass measurements were indexed to body surface area and referenced to age- and sex-based tables.^8^ All CMR data were analyzed using a standardized methodology and analysis package by operators blinded to outcome data.

### Follow-up

The primary outcome was time to first event in a composite of cardiovascular mortality or major HF events. Major HF events comprised heart transplantation, left ventricular assist device implantation, and unplanned HF hospitalization.^14^ An unplanned HF hospitalization was defined as “an event in which the patient was admitted to the hospital with a primary diagnosis of HF, the length of stay was at least 24 hours (or extends over a calendar date), the patient exhibited new or worsening symptoms of HF on presentation, had objective evidence of new or worsening HF, and received initiation or intensification of treatment specifically for HF.”^14^ Changes to oral diuretic therapy did not qualify as initiation or intensification of treatment.^14^ Follow-up data were collected from primary care and hospital records and patient questionnaires. Survival status was also identified using the UK Health and Social Care Information Service to ensure no deaths were missed. Death certificates and post-mortem reports were obtained where applicable. All endpoint events were adjudicated by an independent committee of 3 senior cardiologists with expertise in electrophysiology, HF management, and clinical trial adjudication who were blinded to imaging and genetic data. Endpoints were defined according to the 2014 American College of Cardiology/American Heart Association definitions for cardiovascular endpoints in clinical trials.^14^

Secondary outcomes each consisted of all-cause death, cardiovascular death, major HF events, or major arrhythmic events. Major arrhythmic events comprised sustained ventricular tachycardia, ventricular fibrillation, appropriate implantable cardiac-defibrillator shock, and aborted sudden cardiac death.^14^

### Statistical analysis

Patient characteristics at study enrollment were summarized as median (Q1-Q3) or frequency (%) and were compared between females and males using a Mann-Whitney U test for continuous variables and chi square test or Fisher exact test for categorical variables, with the latter used in cases where cell count <5. Follow-up time was censored at the earliest time of primary outcome, loss to follow-up, noncardiovascular death, or 5 years. Kaplan-Meier (KM) survival curves were generated for the primary outcome and its components and compared using the log-rank test. The proportional hazards assumption was assessed visually using log-log transformed KM plots and Schoenfeld tests and demonstrated clear evidence of a violation for sex (*P* = 0.01). Therefore, univariable and multivariable associations between sex and the primary outcome were investigated using Cox regression models including an interaction term for time to estimate separate HRs comparing females and males during the first 2 years of follow-up and 2 through 5 years follow-up. A set of established factors with prognostic utility in DCM (age, LVEF, and mid-wall myocardial fibrosis) were preselected for inclusion in the multivariable outcome analysis. In sensitivity analyses, NT-proBNP, atrial fibrillation (AF), and NYHA functional class were additionally adjusted for as covariates. To investigate whether these and other DCM prognostic factors (extended to include LA volume, hs-cTnI, NT-proBNP, and left bundle branch block [LBBB]) were modified by sex, tests for interaction were performed. To confirm that the effect of sex on the primary outcome was not dependent on the cut-off time chosen, sensitivity analyses from 0 to 1.5 years and 1.5 to 5 years were performed. Missing covariate data were dealt with using multiple imputation by chained equations with estimates combined over 10 imputation sets using Ruben’s rule. All outcome modelling was performed by expert independent statisticians (R.O., J.G.). All analyses were performed using Stata 17.0.

### Data sharing

Requests for data sharing can be made by contacting the corresponding author. Data will be shared after review and approval by the Cardiovascular Research Centre Science Committee and terms of collaboration will be reached together with a signed data access agreement.

## Results

### Baseline characteristics according to sex

Characteristics of the study cohort at enrollment stratified by sex are shown in Table 1. Study participants were recruited as close as possible to the time of DCM diagnosis (median interval between DCM diagnosis and baseline study CMR scan, which defined enrollment, was 0.1 years [Q1-Q3: 0.0-0.6 years]).

**Table 1:** Baseline Characteristics at Enrollment Stratified by Sex

	Male (n = 398)	Female (n = 206)	*P* Value
Demographic and clinical			
Age, y	53.6 (44.2-64.0)	54.5 (43.6-66.3)	0.38
White	359 (90.2)	165 (80.1)	<0.001
Family history SCD	49 (12.3)	39 (18.9)	0.04
Family history DCM	53 (13.3)	47 (22.8)	0.004
VT	9 (2.3)	2 (1.0)	0.35
NSVT	54 (13.6)	17 (8.3)	0.06
Atrial fibrillation	123 (30.9)	27 (13.1)	<0.001
LBBB	93 (23.4)	71 (34.5)	0.005
Controlled hypertension	115 (28.9)	65 (31.6)	0.51
Diabetes	51 (12.8)	20 (9.7)	0.29
Heart rate, beats/min	71.0 (62.0-85.0)	73.0 (66.0-85.0)	0.12
NYHA functional class			
I	194 (50.5)	58 (30.5)	<0.001
II	147 (38.3)	83 (43.7)	
III/IV	43 (11.2)	49 (25.8)	
Imaging			
LGE mid-wall myocardial fibrosis	166 (41.7)	47 (22.8)	<0.001
LGE extent FWHM, g	9.2 (5.2-17.3)	7.0 (3.6-11.8)	0.09
LGE extent FWHM, %	7.7 (4.9-13.1)	7.8 (4.0-11.7)	0.77
LVEF, %	39 (29-49)	43 (31-52)	0.02
LAVi, mL/m^2^	58 (47-74)	52 (42-66)	<0.001
LVEDVi, mL/m^2^	120 (105-149)	112 (100-134)	<0.001
LVESVi, mL/m^2^	71 (55-103)	64 (49-93)	<0.001
LVSVi, mL/m^2^	48 (38-58)	48 (38-55)	0.47
LVMi, g/m^2^	91 (79-112)	78 (63-93)	<0.001
Medication			
Beta-blockers	283 (71.1)	142 (68.9)	0.57
ACE inhibitor	315 (79.1)	163 (79.1)	1.00
Aldosterone antagonist	141 (35.4)	77 (37.4)	0.66
Diuretic	169 (42.5)	101 (49.0)	0.14
Genetic			
Any variant of	61 (15.3)	30 (14.6)	0.90
*TTNtv*	52 (13.1)	19 (9.2)	0.18
*LMNA*	1 (0.3)	2 (1.0)	0.27
Other sarcomeric	2 (0.5)	1 (0.5)	1.00
Other DCM	3 (0.8)	6 (2.9)	0.07
Biomarkers			
NT-proBNP, pg/mL	437 (116-1478)	556 (139-1361)	0.54
No atrial fibrillation	274 (80-1056)	366 (138-1050)	0.08
Atrial fibrillation	1022 (387-2033)	2000 (825-4206)	0.04
High-sensitivity troponin-I, ng/mL	6.7 (3.2-12.8)	5.0 (2.5-8.6)	0.001
Creatinine, μmol/L	89.0 (78.0-104.0)	75.0 (64.0-89.0)	<0.001
Sodium, mmol/L	146.0 (143.0-150.0)	145.0 (142.0-149.0)	0.51
Potassium, mmol/L	4.6 (4.3-4.8)	4.5 (4.2-4.7)	<0.001
Urea, mmol/L	6.5 (5.4-8.1)	5.9 (4.9-7.6)	0.01
Albumin, g/L	45.0 (42.0-47.0)	43.0 (41.0-46.0)	<0.001
High-sensitivity CRP, mg/L	2.0 (1.0-4.3)	3.2 (1.3-8.3)	0.001
Cholesterol, mmol/L	5.3 (1.3)	5.5 (1.2)	0.53
Urate, μmol/L	415.5 (354.0-517.0)	360.0 (290.0-467.0)	<0.001

Values are median (Q1-Q3) or n (%) and are compared between females and males using a Mann-Whitney U test for continuous variables and chi square test or Fisher exact test for categorical variables, with the latter used in cases where cell count <5. The proportion of missing data for variables heart rate, NYHA functional class, LAVi, LV mass, and biomarkers shown in Supplemental Table 4; the remaining variables had no missingness.

ACE = angiotensin-converting enzyme; CRP = C-reactive protein; DCM = dilated cardiomyopathy; FWHM = full width half maximum; LAVi = left atrial volume index; LBBB = left bundle branch block; LGE = late gadolinium enhancement; *LMNA* = variant in the lamin gene; LVEF = left ventricular ejection fraction; LVEDVi = left ventricular end-diastolic volume index; LVESVi = left ventricular end-systolic volume index; LVMi = left ventricular mass index; NSVT = nonsustained ventricular tachycardia; NT-proBNP = N-terminal pro–B-type natriuretic peptide; SCD = sudden cardiac death; *TTNtv* = truncating variant in the titin gene; VT = ventricular tachycardia.

In total, 604 individuals were enrolled, of whom 206 (34%) were females. Females were more likely to have a history of LBBB at presentation and less likely to have a history of AF. Compared to males, females had higher LVEF (median 43% vs 39%, *P* = 0.02), smaller indexed left ventricular volumes (left ventricular end-diastolic volume index: 112 mL/m^2^ vs 120 mL/m^2^; *P* < 0.001), less commonly had myocardial fibrosis (23% vs 42%), and lower concentrations of hs-cTnI (5 vs 6.7 ng/L, *P* < 0.001) (Table 1) with no difference in age at study enrollment, guideline-directed HF medication use (guideline-directed medical therapy [GDMT]), NT-proBNP concentrations, or extent of myocardial fibrosis. There was no difference in the interval between DCM diagnosis and study enrollment between males (0.1 years, Q1-Q3: 0-0.6 years) and females (0.09 years, Q1-Q3: 0-0.5 years, *P* = 0.87). Despite a generally milder DCM phenotype, females were significantly more functionally limited (higher NYHA functional class on average) than males at presentation (*P* < 0.001).

There was no difference in the overall frequency of DCM genetic variants between males (15.3%) and females (14.6%) (*P* = 0.90) (Table 1), nor was there a specific burden of truncating variants in the titin gene (males 13.1%; females 9.2%; *P* = 0.18).

### Clinical outcome profile in males and females with DCM

**Figure 1:**
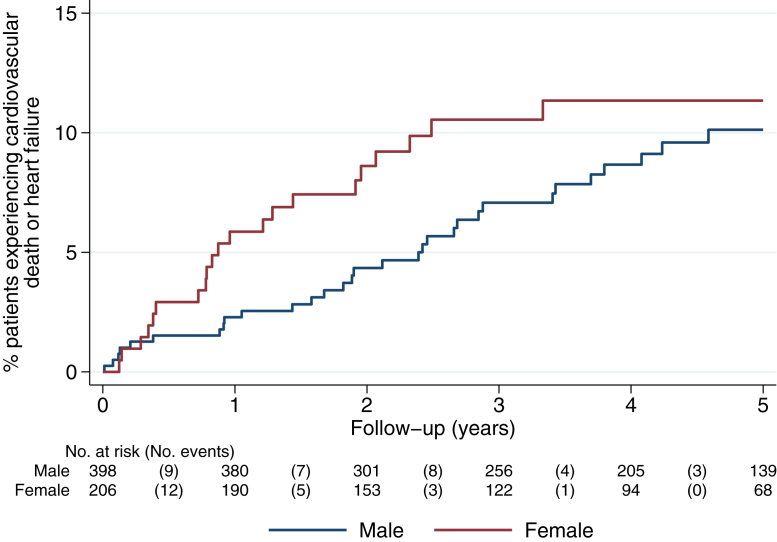
Cumulative Incidence of the Composite of Cardiovascular Death or Heart Failure by Sex Kaplan-Meier plot showing cumulative incidence of the composite outcome of cardiovascular death or heart failure over the study follow-up period (time since enrollment; baseline defined as first diagnostic cardiac magnetic resonance image). Within 2 years, females had more primary outcome events than males. Between 2 and 5 years, males and females had similar outcomes. The numbers in brackets in the risk table are the number of events.

**Figure 2:**
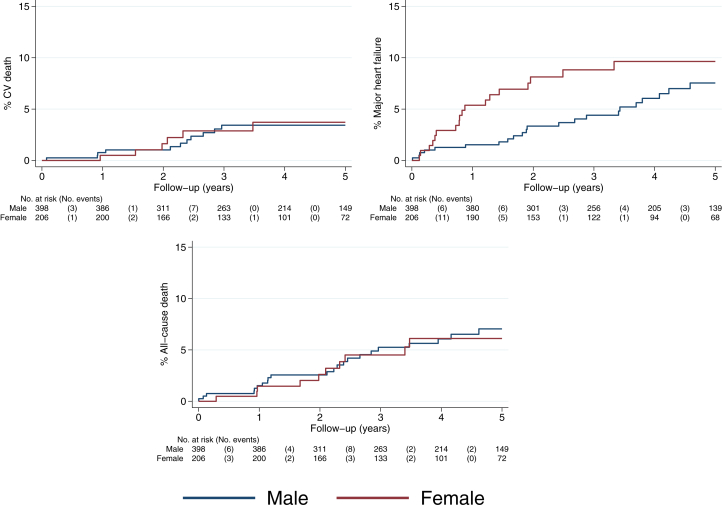
Cumulative Incidence of Cardiovascular Death, Major Heart Failure, and All-Cause Death Kaplan Meier plots showing cumulative incidence of cardiovascular (CV) death, major heart failure events, and all-cause death.

Over 5 years of follow-up (median follow-up time: 3.9 years), there were 52 primary composite events, including 17 cardiovascular deaths and 40 HF events (some study participants had more than one component event) (Supplemental Table 1). There were 15 deaths unrelated to cardiovascular causes. Despite the seemingly favorable baseline profile in females (including much less frequent myocardial fibrosis), within 2 years females had an increased risk of the primary outcome event compared to males (Figure 1). The KM-estimated 2-year event proportions were 8.6% in females vs 4.4% in males (*P* = 0.03) and the unadjusted HR comparing females to males was 2.08 (95% CI: 1.05-4.11). After statistical adjustment for the major DCM prognostic factors of age, LVEF, and mid-wall myocardial fibrosis, the increased risk in females was even more pronounced (adjusted HR: 3.14; 95% CI: 1.55-6.35) (Table 2; full model in Supplemental Table 2). These differences were driven mainly by the rate of major HF events (Figure 2). There was no difference in all-cause death between males and females (Figure 2) or arrhythmic outcomes (Supplemental Table 1). After 2 years, the effect of female sex on prognosis attenuated (between 2 and 5 years adjusted HR: 0.69; 95% CI: 0.63-4.04) (Table 2). The 5-year KM estimates were 10.1% in males and 11.3% in females (Supplemental Table 1).

**Table 2:** Unadjusted and Adjusted HRs for the Primary Outcome Comparing Females to Males Over 5 Years Follow-Up

	Unadjusted		Adjusted^a^	
	HR (95% CI)	*P* Value	HR (95% CI)	*P* Value
Years 0 to 2	2.08 (1.05-4.11)	0.04	3.14 (1.55-6.35)	0.001
Years 2 to 5	0.56 (0.19-1.70)	0.31	0.69 (0.63-4.04)	0.32

a Adjusted for age, LVEF, and LGE. Abbreviations as in Table 1.

### Sensitivity analyses

NT-proBNP concentrations did not differ between males and females at baseline; therefore, NT-proBNP was not included in the primary analysis. However, NT-proBNP is a strong prognostic marker in HF. In this cohort, NT-proBNP concentrations were higher in females compared to males when stratified by AF (Table 1). Therefore, we adjusted for both NTproBNP and AF in sensitivity analyses (Table 3). NT-proBNP was not associated with outcome in this cohort and female sex remained an independent predictor of adverse outcomes at 2 years.

**Table 3:** Sensitivity Analyses of the Primary Outcome Comparing Females to Males Over 5 Years Follow-Up Adjusting for NT-proBNP, AF, and NYHA Functional Class in Addition to the Baseline Model

	Adjusted Variables					
	Age, LVEF, LGE, NT-proBNP (log), and AF		Age, LVEF, LGE, and NYHA Functional Class		Age, LVEF, LGE, NT-proBNP (log), AF, and NYHA Functional Class	
	Adjusted HR (95% CI)	*P* Value	Adjusted HR (95% CI)	*P* Value	Adjusted HR (95% CI)	*P* Value
Years 0 to 2	3.43 (1.63-7.20)	0.001	2.98 (1.42-6.26)	0.004	3.23 (1.49-6.97)	0.003
Years 2 to 5	0.67 (0.21-2.16)	0.51	0.65 (0.20-2.08)	0.47	0.64 (0.20-2.10)	0.46

AF = atrial fibrillation; other abbreviations as in Table 1.

As there were differences in NYHA functional class status in males and females, we assessed whether inclusion of NYHA functional class in the survival model affected the results. After adjusting for NYHA functional class, female sex remained an independent predictor of adverse outcomes at 2 years (Table 3).

The association between sex and primary outcome was not dependent on the cut-off time chosen. Sensitivity analyses from 0 to 1.5 years and 1.5 to 5 years showed that after statistical adjustment for the major DCM prognostic factors of age, LVEF, and mid-wall myocardial fibrosis, females patients with DCM continued to show adverse outcomes between 0 and 1.5 years (adjusted HR: 3.83; 95% CI: 1.72-8.52) (Supplemental Table 3). Similar to the primary analysis, the effect was attenuated after 1.5 years (adjusted HR: 2.12; 95% CI: 0.95-4.70) (Supplemental Table 3).

### Evaluating myocardial fibrosis and LVEF as sex-specific risk factors

**Figure 3:**
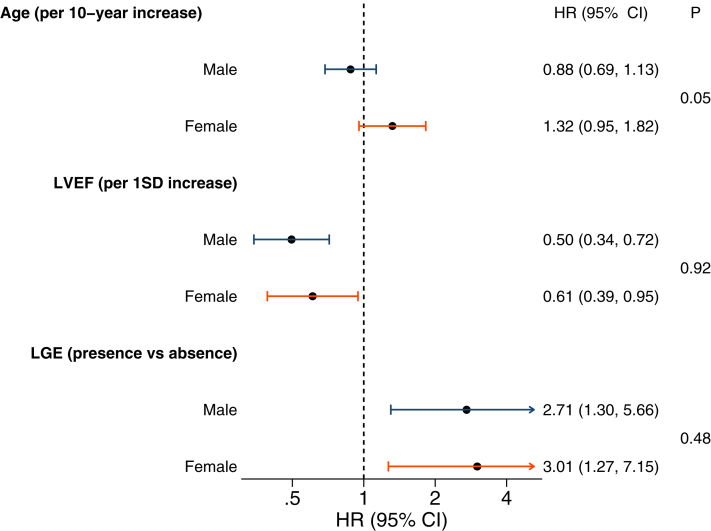
Baseline Variables Associated With the Composite of Cardiovascular Death or Heart Failure Stratified by Sex Forest plot showing the HR for the primary outcome associated with each variable separated by sex. *P* values are calculated from tests for interaction. LGE = late gadolinium enhancement; LVEF = left ventricular ejection fraction.

**Figure 4:**
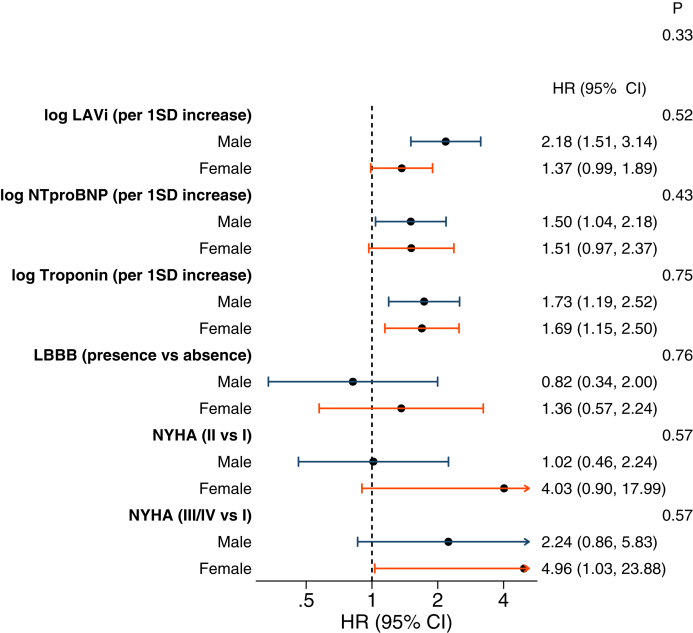
The Association of Other Dilated Cardiomyopathy Prognostic Factors With the Composite of Cardiovascular Death or Heart Failure Stratified by Sex Forest plot showing the HR for the primary outcome associated with each variable separated by sex. *P* values are calculated from tests for interaction. LAVi = left atrial volume index; LBBB = left bundle branch block; NT-proBNP = N-terminal pro–B-type natriuretic peptide.

As female sex was associated with an increased risk of adverse outcomes in adjusted analyses, despite female study participants less commonly having myocardial fibrosis and more likely to have better LVEF, we then explored whether the current most clinically credible predictors of outcome in DCM have similar prognostic value in males and females. We found that mid-wall myocardial fibrosis and LVEF were both associated with outcome in both male and female patients with DCM but neither variable had a significant interaction with sex (Figure 3). Therefore, myocardial fibrosis and LVEF were not sex-specific prognostic factors in this study. Age was also not associated with outcome in either sex. Other potential DCM prognostic factors of LA volume, NT-proBNP, hs-cTnI, LBBB, and NYHA functional class were also not sex-specific prognostic factors (Figure 4). This suggests that additional unrecognized factors contribute to sex-specific risk in DCM.

## Discussion

We identify a novel paradox in prognosis for females with DCM (Central Illustration). This study shows that female sex is an adverse marker in the early natural history of DCM (first 2 years) with a higher risk of major HF events. This was observed despite a seemingly more favorable phenotype at presentation characterized by LV dilatation, higher LVEF, and less prevalent myocardial fibrosis in females compared to males, with no differences in DCM duration, age at presentation, or GDMT. These findings highlight that sex is a relevant prognostic variable in the care of patients with DCM.

**Central Illustration:**
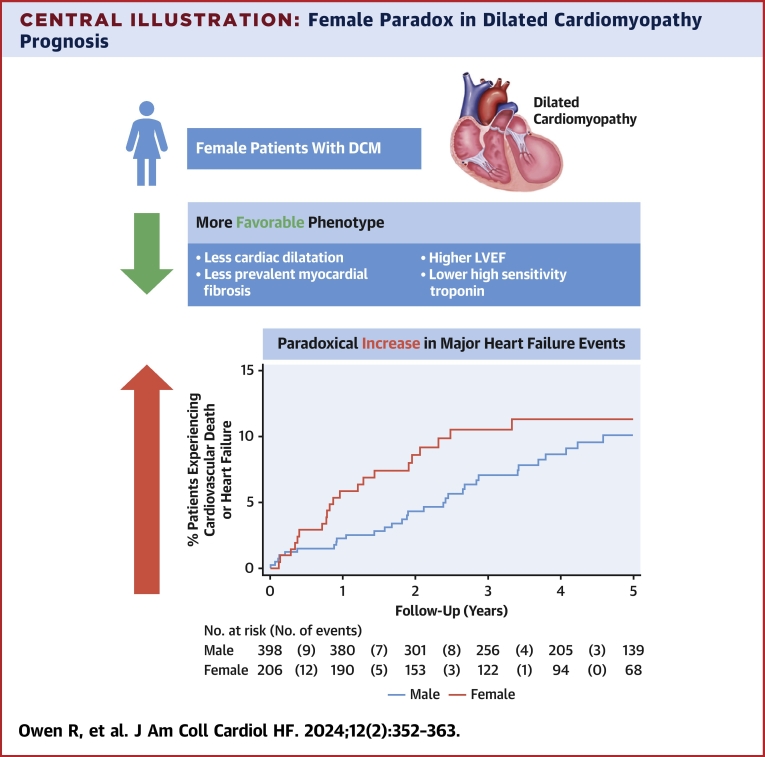
Female Paradox in Dilated Cardiomyopathy Prognosis A novel paradox in prognosis for females with dilated cardiomyopathy (DCM). This study shows that female sex is an adverse marker in the early natural history of DCM (first 2 years) with a higher risk of major heart failure events. This was observed despite a seemingly more favorable phenotype at presentation characterized by less ventricular dilatation, higher left ventricular ejection fraction (LVEF) and less prevalent myocardial fibrosis in females compared to males with no differences in DCM duration, age at presentation, N-terminal pro–B-type natriuretic peptide levels, fibrosis extent, genetic status, or heart failure medication use. These findings highlight that sex is a relevant prognostic variable in the care of patients with DCM.

Despite a worse short-term prognosis, we show that the female DCM phenotype is characterized by much less myocardial fibrosis (23% vs 42%). This is paradoxical, as myocardial fibrosis is one of the strongest prognostic indicators in DCM.^15^ Therefore, a natural extension would be that less prevalent myocardial fibrosis in the female group should be associated with better outcomes. However, female sex was associated with a higher risk of adverse outcomes, even in adjusted analyses. In addition, neither myocardial fibrosis nor LVEF emerged as sex-specific risk factors. Accordingly, other factors responsible for worse short-term outcome in women with DCM are yet to be identified. In line with previous studies, death from cardiovascular causes and major HF events did not change with advancing age and we did not find that age modified sex-specific risk in DCM.^16^ Further work is needed to explore the potential mechanisms for sex-specific risk in DCM, such as the impact of autoimmunity, female reproductive events including pregnancy, and environmental factors, as well as the impact of socioeconomic determinants of health.^17^, ^18^, ^19^

We have shown a marked early elevated risk of major HF events in females with DCM, despite similar intervals since diagnosis and similar HF medication profiles as male patients. This is in keeping with females in our study being more symptomatic at presentation compared to males. These findings indicate a need to better understand factors that may differentially affect individuals affected by DCM to individualize and improve care. HF admissions represent significant morbidity and economic burden, estimated to cost $16,000 per patient per year.^20^ With improvements in GDMT and device therapies, overall mortality is improving in DCM, yet there remains great pressure to improve quality of life and to reduce repeated hospitalizations.^21^ By identifying female sex as an independent risk factor for early adverse outcomes, treatment strategies for patients with DCM can be tailored appropriately (eg, closer monitoring and more aggressive medication titration). Further studies are critically needed to understand how sex influences the deleterious short-term outcome in DCM.

We and others have previously shown that females with DCM experience fewer mortality events (ie, all-cause, cardiovascular, or non–sudden death) compared to men.^16^^,^^22^^,^^23^ However, these older studies lacked granularity in outcome evaluation and these findings can be influenced by competing causes of death. Improvement in mortality rates likely reflects the benefits of contemporary GDMT to lower potential for arrhythmic sudden death. No sex-specific mortality differences were reported in recent major HF or DCM clinical trials (eg, PARADIGM-HF [Efficacy and Safety of LCZ696 Compared to Enalapril on Morbidity and Mortality of Patients With Chronic Heart Failure] and DANISH [Danish ICD Study in Patients With Dilated Cardiomyopathy]) although limitations of these large-scale trials include the small proportion of women.^24^, ^25^, ^26^, ^27^ On the other hand, the results from the current study identify a disconnect between a lower-risk baseline phenotype in women with DCM that is accompanied by a higher propensity toward congestive complications including hospitalization. This paradox may be explained by sex-based differences in what constitutes normal LVEF.^28^ Sex-neutral thresholds are used to define HF syndromes; therefore, a higher LVEF of 50% in a woman with HF symptoms may reflect a relatively greater reduction of systolic function compared with a man with the same LVEF.^29^ Conceptually, women may reverse remodel more with better LVEFs (ie, lowering arrhythmia risk) but remain vulnerable to congestion in part because their sex-adjusted LVEF is still “low” even if the absolute number is higher. Prospective, robust observational data such as from this study are important to our understanding of sex-specific differences in DCM. Our findings share parallels with several other cardiovascular diseases (eg, coronary artery disease and hypertrophic cardiomyopathy) that also show sex-related differences in phenotype expression and outcomes.^30^^,^^31^ The molecular mechanisms underpinning this are complex and are likely to include sex differentially expressed genes, sex hormone effects, and environmental influences. The mechanisms by which biological sex influences DCM disease development and natural history are major targets for further study as indicated by recent guidelines.^5^^,^^29^

### Study limitations

We recognize that biology is not the only determinant of major HF events and there are potential confounders that could contribute to our findings. These include but are not limited to social determinants of health, access to health care, and medication optimization in males vs females during the disease course which were not evaluated in this study. These factors intersect and are critically important to understand as they may respond to specific interventions. For example, social determinants of health such as poor health literacy, which is associated with increased cardiovascular morbidity and mortality, adversely affect women.^19^^,^^32^ Interventions incorporating health literacy can improve therapy adherence and reduce early decompensation in heart failure.^32^ Health literacy may have contributed to the observed sex differences in outcome in this study, and a comprehensive evaluation of the impact of sex disparities in social determinants of health in HF populations is an unmet need. We did not detect differences in genetic disease burden between males and females in this study and were underpowered to detect sex stratified differences in outcome by genotype. Further research in larger populations is underway to evaluate the impact of sex and genotype interactions for DCM prognosis.

## Conclusions

We found that female sex was an adverse modifier of the early natural history of DCM. Females with DCM had a higher early (first 2 years) risk of major HF events driven by congestive complications including HF hospitalization not explained by age, mid-wall myocardial fibrosis, or LVEF at baseline. These findings indicate that sex should be considered an important variable in the care of patients with DCM. Further work is needed to identify sex-specific risk factors and understand the pathophysiological or nonbiological explanations for these sex-specific differences in outcome.

Perspectives **COMPETENCY IN MEDICAL KNOWLEDGE 1:** DCM is associated with cardiovascular mortality, major HF, and arrhythmic events. Female patients with DCM experience more early HF events compared to male patients.

**COMPETENCY IN MEDICAL KNOWLEDGE 2:** The presence of mid-wall myocardial fibrosis is a strong prognostic marker in DCM. Female patients with DCM have less prevalent myocardial fibrosis, but the presence or absence of myocardial fibrosis remains a strong prognostic marker in both males and females with DCM.

**TRANSLATIONAL OUTLOOK 1:** There are clear sex-specific differences in DCM presentation and natural history. Future studies should interrogate the mechanistic basis for these sex differences, including the impact of autoimmunity, female reproductive events including pregnancy, and environmental factors, as well as the impact of socioeconomic determinants of health.

**TRANSLATIONAL OUTLOOK 2:** Although there are sex differences in cardiac morpho-functional characteristics in health and disease, sex-neutral thresholds are currently used to define HF. Future studies should evaluate the sensitivity and specificity of sex-specific diagnostic criteria for HF.

## Funding Support and Author Disclosures

This work was supported by the UK Medical Research Council (MR/W023830/1), the National Heart Lung Institute Research Foundation, Royston Centre for Cardiomyopathy Research, NIHR Biomedical Research Unit Royal Brompton Hospital, NIHR Imperial College Biomedical Research Centre, British Heart Foundation (RE/18/4/34215; SP/10/10/28431; SP/17/11/32885; BH FS/ICRF/21/26019), Wellcome Trust (107469/Z/15/Z), Rosetrees Trust, Sir Jules Thorn Charitable Trust [21JTA], and Alexander Jansons Myocarditis UK. Dr Januzzi has been supported in part by the Hutter Family Professorship. Dr Van Spall has been funded by the Canadian Institutes of Health Research and Heart and Stroke Foundation of Canada. Dr Michos has participated in advisory boards for Novo Nordisk, Novartis, Bayer, Esperion, AstraZeneca, and Amarin. Dr Januzzi has been a trustee of the American College of Cardiology; has been a board member of Imbria Pharmaceuticals; has been a director at Jana Care; has received grant support from Abbott Diagnostics, Applied Therapeutics, HeartFlow, Innolife, and LivaNova; has received consulting fees from Abbott, Bayer, Beckman-Coulter, Boehringer-Ingelheim, Janssen, Novartis, Quidel, Roche Diagnostics, and Siemens; and has participated in clinical endpoint committees/data safety monitoring boards for Abbott, AbbVie, Bayer, CVRx, Intercept, Pfizer, and Takeda. Dr Pennell has received consulting fees from Bayer and Chiesi; has received research support from Bayer and Siemens; and has received speaker fees from Chiesi and Bayer. Dr Cook has been a co-founder and shareholder with Enleofen Bio PTE LTD. Dr Ware has received consulting fees from MyoKardia and Foresite Labs; and has received research support from MyoKardia/Bristol Myers Squibb. Dr Halliday has participated in an advisory board with AstraZeneca. Dr Baruah is working full-time for AstraZeneca. For the purpose of open access, the author has applied a Creative Commons Attribution (CC BY) licence to any Author Accepted Manuscript version arising. All other authors have reported that they have no relationships relevant to the contents of this paper to disclose.
