# Supplementary material for: Sex Differences in the Clinical Presentation and Natural History of Dilated Cardiomyopathy
Source: JACC Heart Fail. 2024 Feb;12(2):352–63. doi: 10.1016/j.jchf.2023.10.009 (PMC10857810; doi:10.1016/j.jchf.2023.10.009)
Supplement: Supplemental Tables 1-5 [file mmc1.docx]

**Supplemental Table 1:** Outcome events at 2 and 5 years stratified by sex shown as counts (Kaplan Meier event proportions).

|  | **N (KM %) at 2 years** | | **P*** | **N (KM %) at 5 years** | | **P**** |
| --- | --- | --- | --- | --- | --- | --- |
|  | **Male** | **Female** |  | **Male** | **Female** |  |
| **Primary outcome** |  |  |  |  |  |  |
| CV death or heart failure | 16 (4.4) | 17 (8.6) | 0.03 | 31 (10.1) | 21 (11.3) | 0.27 |
| **Secondary outcomes** |  |  |  |  |  |  |
| All-cause death | 10 (2.6) | 5 (2.6) | 0.93 | 22 (7.1) | 10 (6.1) | 0.76 |
| CV death | 4 (1.0) | 3 (1.6) | 0.64 | 11 (3.4) | 6 (3.7) | 0.90 |
| Heart failure composite | 12 (3.3) | 16 (8.1) | 0.009 | 22 (7.5) | 18 (9.6) | 0.11 |
| Heart failure hospitalisation | 10 (2.8) | 16 (8.1) | 0.003 | 17 (5.7) | 18 (9.6) | 0.02 |
| LVAD | 3 (0.8) | 0 (0.0) | 0.21 | 5 (1.8) | 0 (0.0) | 0.11 |
| Transplant | 3 (0.9) | 0 (0.0) | 0.21 | 4 (1.3) | 1 (1.4) | 0.51 |
| Arrhythmia composite | 4 (1.0) | 5 (2.6) | 0.18 | 9 (3.1) | 6 (3.3) | 0.62 |
| Stable VT | 3 (0.8) | 5 (2.6) | 0.09 | 8 (2.9) | 5 (2.6) | 0.72 |
| VF | 0 (0.0) | 0 (0.0) | -- | 0 (0.0) | 1 (0.7) | 0.16 |
| ICD | 3 (0.8) | 1 (0.5) | 0.69 | 4 (1.3) | 3 (2.2) | 0.61 |
| Aborted SCD | 3 (0.8) | 1 (0.5) | 0.69 | 4 (1.3) | 3 (2.2) | 0.61 |

*Comparing incident over 2 years using Logrank test

**Comparing incident over 5 years using Logrank test

**Supplemental Table 2.** Full results from the multivariable models assessing the association of sex with the composite of cardiovascular death and heart failure, adjusted for age, LVEF, and LGE

|  | **HR (95% CI)** | **P** |
| --- | --- | --- |
| **0-2 years** |  |  |
| Female | 3.14 (1.55, 6.35) | 0.001 |
| Age (per 10 years) | 0.93 (0.73, 1.17) | 0.52 |
| LVEF (per 10%) | 0.58 (0.43, 0.78) | <0.001 |
| LGE | 3.27 (1.59, 6.74) | 0.001 |
|  |  |  |
| **2-5 years** |  |  |
| Female | 0.69 (0.22, 2.16) | 0.53 |
| Age (per 10 years) | 1.04 (0.74, 1.46) | 0.82 |
| LVEF (per 10%) | 0.71 (0.49, 1.02) | 0.07 |
| LGE | 1.60 (0.63, 4.04) | 0.32 |
|  |  |  |

**Supplemental Table 3.** Sensitivity analysis of the unadjusted and adjusted hazard ratios for the primary outcome comparing females to males over 0 to 1.5 and 1.5 to 5 years follow-up

|  | **Unadjusted** | | **Adjusted*** | |
| --- | --- | --- | --- | --- |
|  | **HR (95% CI)** | **P** | **HR (95% CI)** | **P** |
| **0-1.5 years** |  |  |  |  |
| Female vs Male | 2.66 (1.22, 5.79) | 0.01 | 3.83 (1.72, 8.52) | 0.001 |
| **1.5-5 years** |  |  |  |  |
| Female vs Male | 0.62 (0.25, 1.55) | 0.31 | 2.12 (0.95, 4.70) | 0.07 |

* Adjusted for the following variables: age, LVEF and LGE

**Supplemental Table 4: Missing data.** For variables with missing data, table highlights percentage of missing data. For other variables listed in Table 1 but not shown below, there was no missing data.

|  | **Male (N=398)** | **Female (N=206)** | **P** |
| --- | --- | --- | --- |
|  | **Mean (SD)/n (%)** | **Mean (SD)/n (%)** |  |
| **Demographic & clinical** |  |  |  |
| Heart rate (bpm) | 71.0 (62.0-85.0) | 73.0 (66.0-85.0) | 0.12 |
| *Missing, n (%)* | 17 (4.3) | 16 (7.8) |  |
| NYHA I | 194 (50.5) | 58 (30.5) | <0.001 |
| NYHA II | 147 (38.3) | 83 (43.7) |  |
| NYHA III/IV | 43 (11.2) | 49 (25.8) |  |
| *Missing, n (%)* | 14 (3.5) | 16 (7.8) |  |
| **Imaging** |  |  |  |
| LAVi (ml/m2), median (IQR) | 58 (47-74) | 52 (42-66) | <0.001 |
| *Missing, n (%)* | 15 (3.8) | 9 (4.4) |  |
| LVMi (g/m2), median (IQR) | 91 (79-112) | 78 (63-93) | <0.001 |
| *Missing, n (%)* | 12 (3.0) | 2 (1.0) |  |
|  |  |  |  |
| **Biomarkers** |  |  |  |
| NT-ProBNP, pg/ml, median (IQR) | 437 (116-1478) | 556 (139-1361) | 0.54 |
| *Missing, n (%)* | 70 (17.6) | 53 (25.7) |  |
| High-sensitivity troponin-I, ng/mL, median (IQR) | 6.7 (3.2-12.8) | 5.0 (2.5-8.6) | 0.001 |
| *Missing, n (%)* | 39 (9.8) | 41 (19.9) |  |
| Creatinine, μmol/L | 94 (24) | 79 (23) | <0.001 |
| *Missing, n (%)* | 19 (4.8) | 17 (8.3) |  |
| Sodium, mmol/L | 146.7 (6.1) | 146.6 (6.6) | 0.51 |
| *Missing, n (%)* | 81 (20.4) | 61 (29.6) |  |
| Potassium, mmol/L | 4.6 (0.4) | 4.5 (0.4) | <0.001 |
| *Missing, n (%)* | 135 (33.9) | 85 (41.3) |  |
| Urea, mmol/L, median (IQR) | 6.5 (5.4-8.1) | 5.9 (4.9-7.6) | 0.01 |
| *Missing, n (%)* | 81 (20.4) | 62 (30.1) |  |
| Albumin, g/L | 45 (3.7) | 44 (3.6) | <0.001 |
| *Missing, n (%)* | 82 (20.6) | 62 (30.1) |  |
| High sensitivity CRP, mg/L, median (IQR) | 2.0 (1.0-4.3) | 3.2 (1.3-8.3) | 0.001 |
| *Missing, n (%)* | 81 (20.4) | 62 (30.1) |  |
| Cholesterol, mmol/L | 5.3 (1.3) | 5.5 (1.2) | 0.53 |
| *Missing, n (%)* | 82 (20.6) | 63 (30.6) |  |
| Urate, μmol/L | 440 (118) | 385 (126) | <0.001 |
| *Missing, n (%)* | 84 (21.1) | 63 (30.6) |  |

**Supplemental Table 5: Sex and ancestry.** Event rates according to ancestry and sex. Non- Europeans had a higher rate of the primary outcome whether they were females or males (with a slightly larger difference in males) but this was not statistically significant. There was no evidence that the effect of being European differed according to sex (p for interaction = 0.83).

|  | No. patients | No. events (5y KM %) | HR (95% CI) |
| --- | --- | --- | --- |
| Males |  |  |  |
| Non- European | 39 | 4 (15.6) | 0.66 (0.23, 1.88) |
| European | 359 | 27 (9.6) |  |
| Females |  |  |  |
| Non- European | 41 | 4 (12.8) | 0.77 (0.28, 2.10) |
| European | 165 | 16 (11.0) |  |
